# Body image in idiopathic scoliosis: a comparison study of psychometric properties between four patient-reported outcome instruments

**DOI:** 10.1186/1477-7525-12-81

**Published:** 2014-06-03

**Authors:** Antonia Matamalas, Joan Bagó, Elisabetta D'Agata, Ferran Pellisé

**Affiliations:** 1 Department of Orthopaedic Surgery, Hospital Vall d'Hebron, P Vall d'Hebron, 119, 08035 Barcelona, Spain; 2 Research Institute, Hospital Vall d'Hebron, P Vall d'Hebron, 119, 08035 Barcelona, Spain

**Keywords:** Body image, Idiopathic scoliosis, Patient-reported outcomes

## Abstract

**Background:**

Four patient-reported outcome (PRO) instruments are commonly used to assess body image in idiopathic scoliosis (IS): the Quality of Life Profile for Spinal Deformities (QLPSD), SRS-22 Self-Image scale, Spinal Appearance Questionnaire (SAQ), and Trunk Appearance Perception Scale (TAPS). The aim of this study is to compare the psychometric properties of these instruments in patients with IS and report the translational/cultural adaptation of the SAQ to Spanish.

**Methods:**

The four instruments in a Spanish version were administered to 80 patients with IS aged 10 to 40 years old. The sample was stratified according to scoliosis magnitude (less and more than 45º). Analysis was also conducted for age groups. The psychometric properties studied included convergent and divergent construct validity, as well as internal consistency. Convergent validity was evaluated by correlation analysis between the self-image instruments and Cobb angle. Divergent validity was assessed with correlation analysis between PRO scores and SRS-22 dimensions scores such as Function, Pain and Mental Health.

**Results:**

In the overall sample, each of the PRO instruments demonstrated high internal consistency (QLPSD Body Image, α = 0.80; SRS-22 Self Image, α = 0.78; SAQ, α = 0.89; TAPS, α = 0.87), also both for younger and adult patients subgroups. Correlation with curve magnitude was significant for each of the four scales. However, the correlation was higher for the pictorial scales (SAQ Appearance r = 0.61, TAPS r = - 0.62) than for the textual scales (QLPSD-bi r = 0.36, SRS-22 Self-Image scale r = - 0.41). In the younger group, correlation between Cobb angle and textual scales (QLPSD-bi and SRS-22 Self-Image Scale) was not significant. Body Image scales showed significant correlations with SRS-22 Pain, Function and Mental Health dimensions.

**Conclusions:**

All four instruments tested have good psychometric properties. Pictorial scales (SAQ Appearance and TAPS) correlated better with the radiological magnitude of the curve and this correlation is independent of age. Unexpectedly, all four scales demonstrated significant correlations with non-body image dimensions and the divergent hypothesis was not confirmed. Globally, pictorial scales showed slightly better construct validity to test body image perception than textual scales.

## Background

The value of patient-reported outcome (PRO) measures for improving the care and satisfaction of patients is now well established [1]. Cosmetic impairment is noteworthy in persons with idiopathic scoliosis (IS). Consequently, perceived body image is an important factor when assessing health-related quality of life (HRQOL) in those individuals [2]. The perception of body image in IS has been evaluated by various PRO instruments. In chronological order of publication and according to the available published information, the most frequently used are the Quality of Life Profile for Spinal Deformities (QLPSD) [3], the SRS-22 Patient Questionnaire Self-Image subscale [4-6], the Spinal Appearance Questionnaire (SAQ) [7-9], and the Trunk Appearance Perception Scale (TAPS) [10].

The QLPSD was developed in Spanish [3] to assess HRQOL in adolescents with IS. The questionnaire contains 21 items grouped in 5 dimensions: psychosocial functioning, sleep disturbances, back pain, back flexibility, and body image. Body image subscale internal consistency was found to be adequate for clinical research (Cronbach’s alpha = 0.7) as was test-retest reliability (ICC = 0.66). However, there was no significant correlation between the scale score and Cobb angle.

SRS-22 was designed by Asher et al. [4-6] for the outcome assessment of patients with IS. The SRS-22 consists of 22 items belonging to 5 dimensions: Function/Activity, Pain, Self-Image, Mental Health, and Satisfaction with Treatment. Adequate internal consistency (Cronbach’s alpha = 0.7) and reproducibility (ICC = 0.9) were found for the Self-Image Subscale whereas correlation with Cobb angle was statistically significant (r = - 0.5).

The SAQ is a pictorial scale based on the Walter Reed Visual Assessment Scale (WRVAS) [11]. The test measures the patients’ perception of their deformity through a scale based on drawings of the body. It has been tested in adolescents with IS. In the first version of the SAQ, designed by Sanders et al. [7], the WRVAS was refined by adding several drawings and a second scale regarding expectations about body image. This first version consisted of 32 questions in 9 domains. With the use of factor analysis, Carreon et al. [8] recently found that 14 questions were associated with two factors: 10 were linked to a scale of appearance (SAQ Appearance) and 4 to a scale of expectations (SAQ Expectations). The reported internal consistency for the total score was 0.88 and the test-retest reliability was 0.89. Nevertheless, correlation between the total score and the Cobb angle was only 0.32.

Finally, TAPS was originally designed in Spanish in order to assess patient perception of trunk deformity in individuals with IS [10]. Cronbach's alpha coefficient was 0.89 and the ICC for the mean sum score to assess test-retest reliability was 0.92, whereas correlation between TAPS mean score and Cobb angle was -0.55.

These four instruments have been separately evaluated in disparate situations, such as with different age groups, treatments, or curve magnitudes. These circumstances could explain the above-mentioned differences found. The final goal of using these instruments is to evaluate the effect of different treatment modalities into patients’ body image perception, in addition to the radiological (Cobb angle) and HRQOL evaluation (SRS-22 Patient Questionnaire is the standard instrument used for this purpose).

As clinicians, we want to know which of the above-mentioned instruments may be better in evaluating patients in our daily practice. We are especially interested in analyzing the relationship between the instrument scores and the curve magnitude, because the Cobb angle is generally recognized as the gold standard measure of disease’s severity. Moreover, we wanted to determine the relationship between these four instruments and the other HRQOL dimensions, such as pain, mental health, and function.

The aim of this study is to compare the psychometric properties (internal consistency and construct validity) of these four instruments in a single group of patients with IS. In addition, we will present the cross-cultural adaptation of the SAQ into Spanish.

## Methods

This is a cross-sectional study, approved by the Clinical Research and Ethics Committee of our hospital. The inclusion criteria were patients with IS, 10 to 40 years old, who had not received previous surgical treatment and who agreed to participate in the study. For each patient, posterior-anterior full-length radiographs were performed one week before participation. An orthopedic surgeon (AM) performed all angle measurements using Surgimap Spine Software (Nemaris Inc, New York, NY). For the analysis, the magnitude of the curve with the largest Cobb angle (MLC) of all the patient’s curves was used. Only those patients that had a MLC ≥ 25° in the coronal plane were included. This threshold was chosen because it is generally accepted that curves below 25° do not need any treatment [12].

The sample was stratified according to MLC in two groups: Group <45° and Group ≥ 45°. This cut-off value of 45° was chosen because at this magnitude, surgical treatment is usually recommended [12]. We calculated that each group should be comprised of 40 patients in order to obtain a significant between-groups difference in the TAPS score, according to the previously reported data [10]. Patients were recruited consecutively until the required number for each group was obtained.

All patients completed the SRS-22, QLPSD Body Image Scale, SAQ and TAPS questionnaires on the day of the visit. Questionnaires were administered using paper-based forms and they were completed by the patients themselves without any assistance of the attending physician or of the patients’ parents before the consultation. The researcher who measured x-rays was unaware of questionnaire scores.

### Outcome instruments

#### Quality of life profile for spinal deformities body image scale (QLPSD-bi)

The QLPSD-bi evaluates body image in adolescents with IS and includes 4 items. Patients had to rate their agreement or disagreement with each statement on the questionnaire using a five-point Likert scale. The total score of the domain ranges from 4 (best perception) to 20 (worst perception). In this study, we used the original Spanish version of the instrument [3].

### Scoliosis research society-22

The SRS-22 consists of 22 items belonging to 5 dimensions: Function/Activity, Pain, Self-Image, Mental Health, and Satisfaction with Treatment. Each domain had five items each, with the exception of satisfaction with treatment, which had two items. The two satisfaction items were not included in the final analysis. Each question is answered using a five-point Likert scale ranging from 1 (worst) to 5 (best). Results are presented as the mean of each scale (sum of 5 questions/5) and the mean subtotal score (sum of 20 questions/20); hence, ranking ranges are from 1 to 5. In this study, we used the validated Spanish version of the instrument [13].

### Spinal appearance questionnaire (SAQ)

SAQ consists of two parts: SAQ Appearance and SAQ Expectation.

14 questions were associated with two factors: 10 were linked to a scale of appearance (SAQ Appearance) that measures patient’s perception of spinal deformity’s appearance; and 4 to a scale of expectations (SAQ Expectations) which measures expectations about Self Image.

The SAQ has a total possible score ranging from 14 (best score) to 70 (worst score). The scale is composed of two domains. The SAQ Appearance domain is based on 10 drawings with a score of 1 (best score) to 5 (worst score) and a possible range of 10 to 50. The Expectations domain is comprised of a five-point Likert scale with 4 items, with a total sum ranging from 4 (lower expectations) to 20 (higher expectations) [8,9].

For the present study, we first performed a transcultural adaptation of the SAQ items from the original English into Spanish. The cross-cultural adaptation process was performed using the guidelines of the International Quality of Life Assessment (IQOLA) Project [14,15]. Starting with the original English version, two independent translators each produced a translation into Spanish. Two other independent translators then translated the SAQ back into English. The first two of the translators were native English speakers and the last two were native Spanish speakers. An expert committee that was comprised of the translators, one spine surgeon, one specialist in physical medicine, and one psychologist specializing in spine deformities assessed the translations. A final version was developed by consensus of the entire working group (Additional file 1).

### Trunk appearance perception scale (TAPS)

The TAPS includes 3 sets of drawings, corresponding to 3 viewpoints of the trunk: looking towards the back, looking towards the head with the patient bending over, and looking towards the front. The last drawing has two sets, one for women and one for men. Each drawing is scored from 1 (greatest deformity) to 5 (least deformity), and a mean total is then obtained, with results ranging from 1 to 5. On this scale, patients have to choose the drawings that are most similar to their perception of their body image. The original Spanish version of the test was used for the current study [10].

### Analysis

SPSS 17.0 software was used for the statistical analyses. We included all data that were obtained for all patients, as no missing data were found upon final review. In the descriptive analysis, the mean and standard deviation (SD) were calculated for all variables. Data were analyzed separately according to the age groups. Mean differences were assessed with a Student t-test. Reliability of the outcome instruments was estimated by the internal consistency and it was determined using Cronbach's alpha coefficient. We have considered as acceptable a value of Cronbach's alpha ranging from 0.7 to 0.95 (Tavakol and Dennick, 2011) [16]. Reliability was assessed both for the entire sample and for each age group (younger and older than 18 years old).

We hypothesized that the PRO instrument scores were correlated with the magnitude of the curve. Consequently, the mean PRO instrument score should be different between the two groups of different curve magnitude. To test this hypothesis we first calculated the Pearson’s correlation coefficient between MLC and PRO instruments scores. We then conducted a Student’s t-test to analyze mean difference between MLC groups. Secondly, we hypothesized that the scales evaluating body image would correlate strongly (i.e., correlation coefficient > 0.6) between them but they would not correlate (i.e. correlation coefficient < 0.3) with other dimensions such as mental health, pain or function. To test these hypotheses, we determined the inter-correlations by finding the Pearson’s correlation coefficient between the image scales (QLPSD_bi, SRS-22 image; SAQ and TAPS) and the correlations among these scales and mental health, pain and function SRS-22 scales. In addition, data were also analyzed separately for the two age groups. Statistical significance was set at p < 0.05.

## Results

The study included 80 patients, with a mean age of 20.3 years (range 10–40 years), 85% of which were females. 40 patients belong to group < 45° as well as to group ≥ 45°. The average MLC was 45.9° (range 25.1°–77.2°): group < 45° averaged 35.2° (range 25.1º–44.2°) and group ≥ 45° averaged 56.6° (range 45°–77.2°). Mean scores of the different outcome instruments were as follows: QLPSD-bi mean score 11.42 (range 4–20) and the mean SRS-22 Self-Image score was 3.2 (range 1.4–5). The SAQ total mean was 39.6 (range 14–61). The SAQ Appearance mean was 24.27 (range 10–42). The SAQ Expectations mean was 15.3 (range 4–20). The average TAPS value was 3.2 (range 1–5).

### Internal consistency

Internal consistency (Cronbach’s alpha) was satisfactory for all of the scales: QLPSD-bi α = 0.80, SRS-22 Body Image α = 0.78, SAQ Total α = 0.88, SAQ Appearance α = 0.89, SAQ Expectations α = 0.87, and TAPS α = 0.87. In Table 1, Cronbach’s alpha coefficients for the current study and the original reports are detailed.

**Table 1:** Crombach’s alpha coefficients in the original reports and current study

**Instrument**	**Internal consistency**	
	**Original**	**Current**
**QLPSD-bi**	α = 0.70^3*^	α = 0.80
**SRS-22 self image**	α = 0.73^13*^	α = 0.78
**SAQ total**	α = 0.88^8*^	α = 0.88
***SAQ appearance***	α = 0.89^8*^	α = 0.89
***SAQ expectations***	α = 0.88^8*^	α = 0.87
**TAPS**	α = 0.89^10*^	α = 0.87

*Bibliographical references from which the original values were found.

### Construct validity

#### Correlation between outcome instrument scores and the radiological magnitude

The MLC showed a significant correlation with the QLPSD-bi score (r = 0.36; p < 0.05) and the SRS-22 Self-Image scale (r = - 0.41, p < 0.05). Correlation between the MLC and SAQ scales was: SAQ Total r = 0.55 p < 0.05); SAQ Appearance r = 0.61 (p < 0.05) and SAQ Expectations r = 0.24 (p < 0.05). TAPS significantly correlated with the MLC (r = - 0.62, p < 0.05). In addition, we analyzed the mean score differences between the MLC groups. As Table 2 shows, the group ≥ 45° was found to have significantly worse body image perception across all scales significantly, with the exception of SAQ expectation.

**Table 2:** Mean scores of PRO Instruments according to curve magnitude groups

**Instrument**	**Group**	**Mean (SD)**	**P-value (Student *t *** **test)**
**QLPSD-bi**	< 45°	10.4 (3.6)	p = 0.05
	≥ 45°	12.4 (5)	
**SRS-22 self image**	< 45°	3.43 (0.6)	p = 0.007
	≥ 45°	3 (0.8)	
**SAQ total**	< 45°	34.8 (9.3)	p < 0.001
	≥ 45°	44.3 (9.2)	
**SAQ app**	< 45°	20.33 (5.6)	p < 0.001
	≥ 45°	28.18 (6.7)	
**SAQ expect**	< 45°	14.5 (5.2)	p = 0.13
	≥ 45°	16.2 (4.5)	
**TAPS**	< 45°	3.8 (0.7)	p < 0.001
	≥ 45°	2.7 (0.7)	

#### Correlation among outcome instruments

To assess the convergent-divergent validity, we determined the inter-correlations among the four instruments and the correlations between the instruments and mental health, pain and function SRS-22 scales (Additional file 2). Body image instruments showed a significant correlation among each of them (Table 3). The direction of correlations was correct considering that TAPS and SRS-22 scoring is the inverse of SAQ and QLPSD-bi scoring. We highlight the correlations between TAPS and SAQ Appearance (r = -.80) and between SRS-22 Self-Image and QLPSD-bi (r = - 0.76). It is also worth noting the lower correlations that were observed between the SAQ expectation scale and the other instruments ranged in absolute magnitude in the expected directions.

**Table 3:** Pearson correlations among all the scales assessed for the overall sample

	**SAQ app**	**QLPSDbi**	**Self-image SRS-22**	**SAQ expect**	**Function SRS22**	**Pain SRS22**	**Mental health SRS22**
TAPS	-.80**	-.35**	.46**	-.36**	.50**	.42**	.27*
SAQ.App		.60**	-.67**	.42**	-.6**	-.49**	-.43**
QLPSD-bi			-.76**	.61**	-.43**	-.20	-.45**
Self-Image SRS-22				-.55**	.68**	.50**	.60**
SAQ.expect					-.29**	-.24*	-.20
Function srs-22						.70**	.56**
Pain SRS-22							.52**

*α < 0.05.

**α < 0.01.

SRS-22 Function scale correlated significantly with the image scales, with coefficients ranging in absolute magnitude from -0.3 to 0.68 in the expected directions. SRS-22 Pain scale also correlated with image scales except with QLPSD-bi, with coefficients ranging in absolute magnitude from -0.24 to 0.49 in the expected directions. Finally, SRS-22 Mental Health scales correlated with the image scales, with coefficients ranging from 0.27 to 0.58. SAQ Expectation correlated moderately with the image scales, with coefficients ranging in absolute magnitude from 0.6 to -0.36 in the expected directions, and correlated weakly with mental health, pain (r = - 0.2) and function (r = 0.3) SRS-22 domains. The data did not support our hypothesis because the correlations that are significant were expected to be non-significant and weaker under the divergent hypotheses tested.

### Analysis by age group

The sample was comprised of 42 patients under 18 years old (average 13.9 years) and 38 patients older than 18 years of age (average 27.3 years). In Table 4, data are summarized, including sex and MLC, for the overall sample and for each age group. For each instrument and each age group, data concerning the mean scale score, internal consistency, and correlation coefficient between the scale and MLC are presented. Scoliosis magnitude was somewhat larger in the older group and the sex distribution was similar between age groups. Mean instrument scores were significantly worse in the older group, with the exception of the SAQ Expectations scale. Internal consistency was similar in both groups. The correlation between the MLC and instrument scores was similar in both groups for pictorial scales, but it was remarkably different for textual scales. The older group showed stronger correlations than did the younger group.

**Table 4:** Data on age, MLC, gender and PRO instruments (mean score, internal consistency and correlation with MLC)

	**< 18 years old**	**≥ 18 years old**	**Overall sample**
n	42	38	80
Mean age (years) (SD)	13.9 (1.9)	27.3 (7.4)	20.3 (8.6)
MLC (SD)	42.8° (13.0)	49° * (11.7)	21.2° (13)
Gender (% female)	85.7%	84.2%	85%
**TAPS**			
Mean score (max score = 5) (SD)	3.6 (0.8)	2.8* (0.8)	3.2 (.9)
Internal consistency (Cronbach’s α)	.83	.85	.87
Relation with Cobb angle (Pearson’s r)	-.64	-.55	-.62
**Appearance SAQ**			
Mean score (max score = 50) (SD)	21.3 (6.6)	27.6* (6.8)	15.3 (7.3)
Internal consistency (Cronbach’s α)	.86	.87	.89
Relation with Cobb angle (Pearson’s r)	.6	.55	.61
**Expectation SAQ**			
Mean score (max score = 20) (SD)	15.3 (4.8)	15.4 (5.0)	24.3 (5.0)
Internal consistency (Cronbach’s α)	.84	.9	.87
Relation with Cobb angle (Pearson’s r)	.26^**^	.23^**^	.24
**QLPSD-bi**			
Mean score (max score = 20) (SD)	10.8 (4.0)	12.11* (4.9)	11.4 (4.4)
Internal consistency (Cronbach’s α)	.8	.86	.8
Relation with Cobb angle (Pearson’s r)	.2^**^	.47	.36
**SRS-22 Self Image**			
Mean score (max score = 5) (SD)	3.4 (3.1)	2.98* (3.8)	3.4 (3.6)
Internal consistency (Cronbach’s α)	.73	.81	.78
Relation with Cobb angle (Pearson’s r)	-.2 ^**^	-.55	-.41

*Indicates significant between-groups mean differences in t-Student test.

**Indicates no significant correlation coefficient.

## Discussion

Overall, the four scales have good psychometric properties, including adequate internal consistency, fair correlation with scoliosis magnitude, and significant inter-correlation between the four scales. These instruments also showed a significant correlation with the non-image dimensions of pain, daily function, and mental health. Consequently, our hypotheses regarding the divergent validity of the instruments were not supported by the results. In particular, all of the tests showed satisfactory internal consistency (> 0.7), especially the pictorial scales: SAQ Appearance (α = 0.89) and TAPS (α = 0.87). To analyze the construct validity of instruments, we assessed convergent and divergent validity. The convergent validity was analyzed in two ways. First, the correlation between the instrument score and the MLC was determined. The highest correlation coefficients were between the MLC and the pictorial scales (TAPS r = 0.62, SAQ Appearance r = 0.61); textual scales showed significant but moderate correlation with the MLC (SRS-22 Self-Image scale r = - 0.41, QLPSD-bi score r = 0.36), whereas the weakest coefficient was obtained for SAQ Expectations (r = 0.24). To confirm this relationship, we also determined the instrument mean score differences between groups of curves above and below 45°. Patients with curves greater than 45° were found to have the worst scores across all instruments, except for the SAQ Expectations. Our data supports the findings of previous research. Worst scores in greater curves have been reported for SAQ [7], TAPS [10] and SRS-22 [17].

Secondly, correlations among the four instruments were performed. All scales were significantly correlated. The highest correlations were found between TAPS and SAQ Appearance (r = - 0.8), as well as between QLPSD-bi and SRS-22 Self-Image (r = - 0.75). These data indicated that the four scales explore the same dimension. Nevertheless, pictorial scales had a higher correlation between them than the textual scales had. This finding may either suggest that pictorial and textual scales may assess slightly different constructs within the same body image dimension, or that some of the association is due to differences in the scale format (textual versus pictorial).

Before testing the divergent validity, we hypothesized that body image perception instruments would not correlate with instruments measuring other dimensions, such as pain, daily function, and mental health. We evaluated these dimensions using the SRS-22 subscales. We hypothesized that there would be low correlations between the body image scales and the other dimensions. However, the correlations were significant and ranged in absolute magnitude from r = - 0.80 to r = 0.68 in the expected directions (Table 3). They were the highest for the SRS-22 Self-Image subscale, but some correlations over 0.5 were also observed for both the TAPS and the SAQ. These data confirm that perceived body image is a prominent constituent in HRQOL of patients with scoliosis. The results also found that the body image scales have modest divergent validity, with pictorial scales having a lesser correlation with the non-body image dimensions.

Analysis by age groups was also performed. We chose 18 years as the cut-off value because it is usually the age required to include patients in “adult” scoliosis registries. Internal consistency was similar in both groups. However, the mean instrument scores were significantly worse in the older group than in the younger group. Our data supported the similar findings previously reported for TAPS [10] and SRS-22 [18]. The correlation between the MLC and instrument scores was similar in both age groups for the pictorial scales, but it was remarkably different when using the textual scales. In the younger group, there was a lack of correlation between the textual scales score and MLC. This finding calls into question the validity of the textual body image scales when used with younger patients. Parent et al. [18] have mentioned similar limitations with using the SRS-22 questionnaire in this age group, where ceiling effect is also remarkable. Nevertheless, a deeper analysis is warranted because we have not considered other co-variables that may influence body image perception in younger patients.

In this study, we used the Spanish versions of the various assessment tools. The QLPSD-bi [3] and TAPS scale [10] were originally created in Spanish, and a properly validated Spanish version of the SRS-22 is available [13,19]. However, when the study was designed, there was no Spanish version of the SAQ. Therefore, we first performed a cross-cultural adaptation of the instrument, using previously recommended methods [14,15]. Comparisons of the psychometric properties of the various instruments calculated in our study with those of the original versions are shown in Table 1. When considering the internal consistency, the values between the two sets of data are very similar [3,7,10,13].

The SAQ Expectations domain is a novel, unique scale that evaluates patients’ expectations regarding scoliosis surgery. Although its internal consistency is satisfactory, it has very low correlation with MLC. When the Expectation scale is added to the Appearance scale, a paradoxical effect occurs, because the correlation with MLC of the full scale is lower than that of the Appearance scale alone. A patient’s expectation is a complex concept that is difficult to define, measure, and analyze. There is no unanimous agreement on the suitability of an instrument to assess patients’ expectations [20,21]. The SAQ Expectations scale assesses the desire to improve several cosmetic aspects related to the condition. However, some patients who undergo surgery mention other expectations, such as decreasing pain or maintaining satisfactory physical function, in addition to improving body image [22]. A significant relationship has not been found between patient expectations and the actual change in symptoms or the overall satisfaction with treatment outcomes [20]. These considerations make us doubt the advisability of adding an expectations scale to one of the body image perception scales.

The SAQ has some limitations that should be considered. There are many different versions available [7,8]. The first one included 20 items, including eight pictorial items related to deformity and 12 questions on the patient’s expectations regarding treatment. A second version (SAQ v 1.1) [7] was then created containing 33 items: 11 pictorial items and 22 questions on the expectations regarding treatment. However, factor analysis [8] demonstrated that only 14 items aggregated in two factors: 10 items in an “appearance” factor and 4 items in an “expectation” factor. The final instrument shows satisfactory internal consistency and test-retest reliability. However, the above-mentioned paper [8] includes several mistakes especially with regard to the scoring of the two subscales. These errors were amended and published in a subsequent paper [9]. Nevertheless, it is still unclear whether the version 33 items version or the 14 items version is the one recommended by the authors. For our research we decided to use the 14 items version based on its better factorial structure. The internal consistency and divergent validity of SAQ Appearance and TAPS are very similar. As the SAQ Appearance scale is longer and adolescents may have some difficulty with understanding the drawings [23], we suggest the TAPS may be more usable in daily practice. It is a very short form, with only three pictorial items, and it is quick and easy to complete. SRS-22 Self-Image and QLSDP-bi have similar properties. Nevertheless, only the Spanish version of the QLSDP has been validated, whereas SRS-22 has been translated into several languages.

In this research, we have only evaluated how age and scoliosis magnitude influence body image perception scales. Nevertheless, we have not examined the influence on the body image scales of the other factors, such the type of treatment or surface disfigurement measurements, which have been identified as influencing one’s body image perception [19,24].

Finally, we would like to point out that an important aspect in any PRO instrument that should be examined when the instrument is used for evaluative purposes is the instrument’s responsiveness to the changes associated with a therapeutic intervention. Responsiveness after surgical treatment of scoliosis has been reported separately for SRS-22 [5,25,26], SAQ [7], TAPS [27], and QLPSD [28].

Nonetheless, this analysis was not an objective of the current study. In the future, it would be interesting to determine the responsiveness of the four instruments face-to-face in the same group of patients and using different treatment modalities, before making a clinical recommendation for longitudinal studies.

## Conclusions

Overall, the four scales have good psychometric properties, including internal consistency, correlation with scoliosis magnitude, and inter-correlation between the four scales. These instruments showed significant correlation with non-image dimensions, which did not support our hypotheses regarding the divergent validity of the instruments. Pictorial scales (SAQ Appearance and TAPS) correlated better with radiological magnitude of the curve and this correlation was found to be independent of age. However, the correlation between textual scales (QLSDP Body Image and SRS-22 Self-Image scale) and the magnitude of the curve was low in younger patients. Pictorial scales presented a lower correlation with the HRQOL domains, with the exception of self-image, as compared with the textual scales. Globally, pictorial scales showed slightly better construct validity to test body image perception than did the textual scales. Considering that body image is a multidimensional construct, we think it is better evaluated with concurrent use of both pictorial and textual scales.

## Competing interests

The authors declare that they have no competing interests.

## Authors’ contributions

AM, JB contributed to conception and design the study. AM, ED participated in acquisition of data. AM, JB, ED contributed to literature review, analysis and interpretation of data. JB, ED participated in writing the article. FP revised the final version. All authors read and approved the final manuscript.

## Supplementary Material

Additional file 1 SAQ Spanish version. Click here for file

Additional file 2 Pearson’s correlations among all the scales assessed for the two groups. Click here for file
