# Supplementary material for: Body image in idiopathic scoliosis: a comparison study of psychometric properties between four patient-reported outcome instruments
Source: Health Qual Life Outcomes. 2014 Jun 3;12:81. doi: 10.1186/1477-7525-12-81 (PMC4049402; doi:10.1186/1477-7525-12-81)
Supplement: Additional file 1 — SAQ Spanish version. [file 1477-7525-12-81-S1.pdf]

APPENDIX 1

Spanish version of SAQ

SAQ

Por favor, observe atentamente las imágenes siguientes, que muestran distintas formas de la columna. Marque el círculo debajo del dibujo que más se parezca a usted.

SAQ Apariencia

Curva del cuerpo

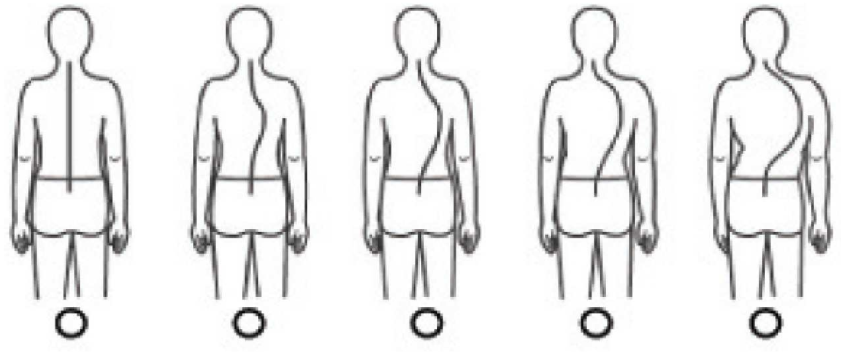

Prominencia de las costillas

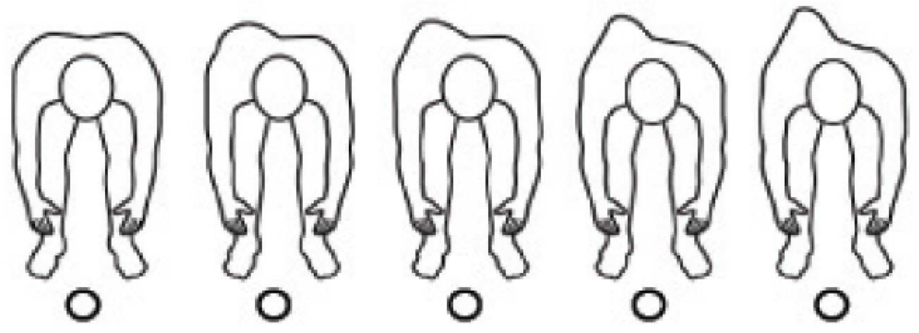

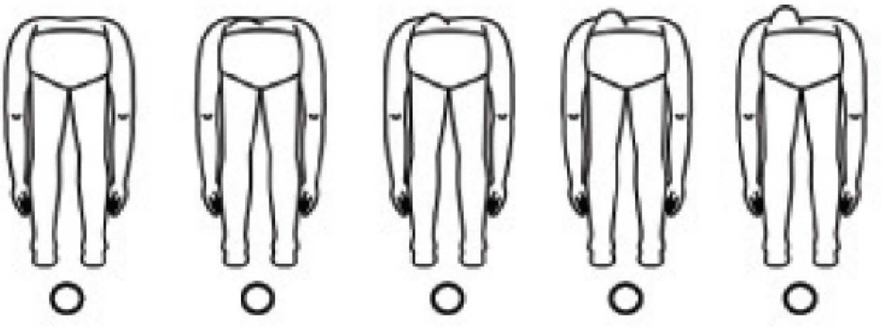

Prominencia lumbar

Cabeza-torax-caderas

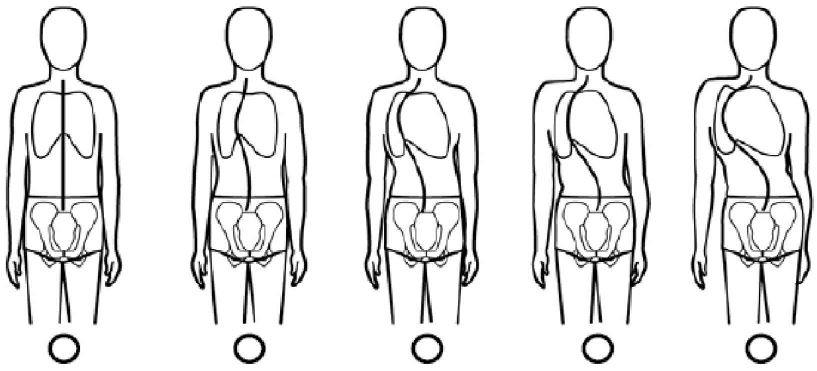

Posición de la cabeza en relación con las caderas

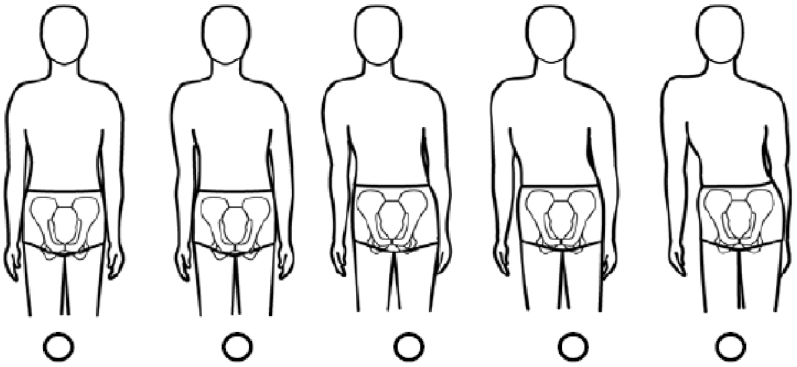

Nivel de los hombros

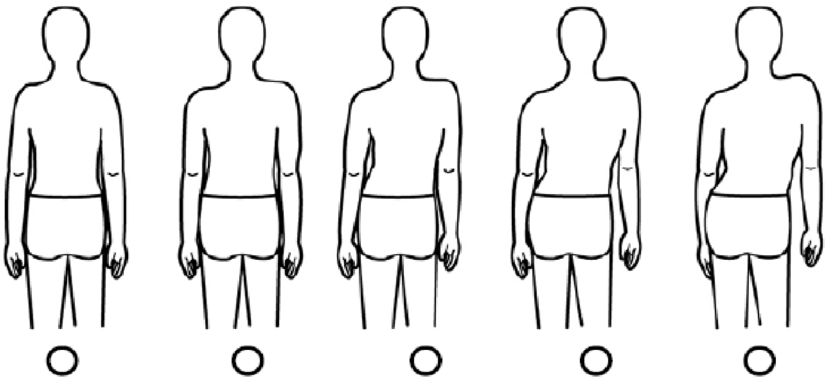

Rotación de los omoplatos

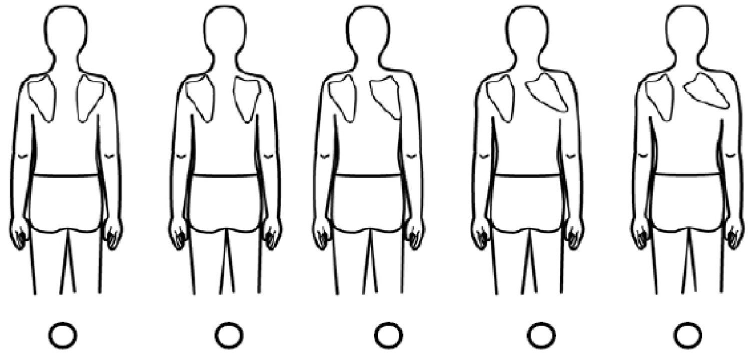

Angulo de los hombros

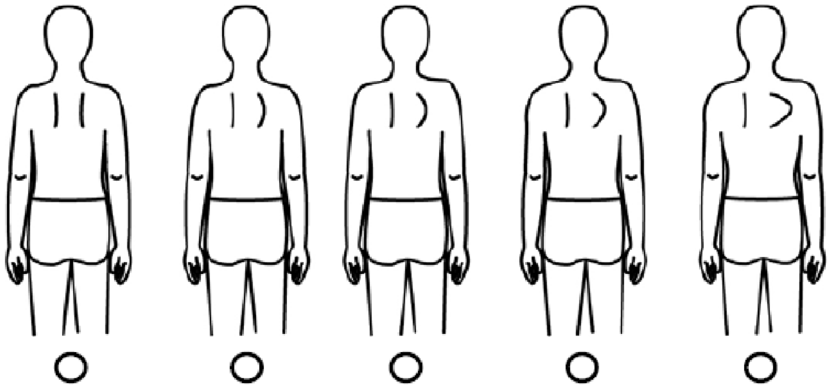

Posición de la cabeza

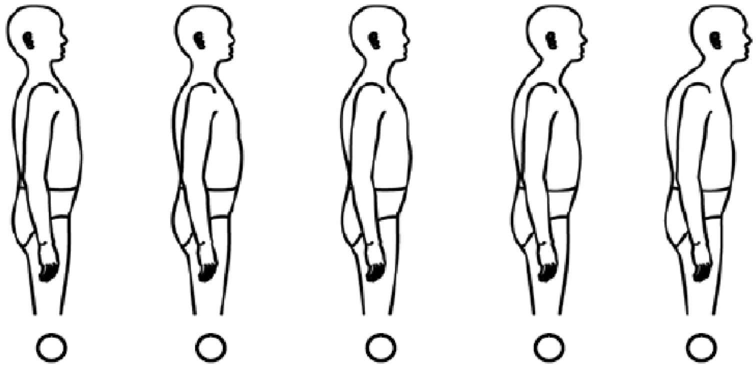

Prominencia de la columna

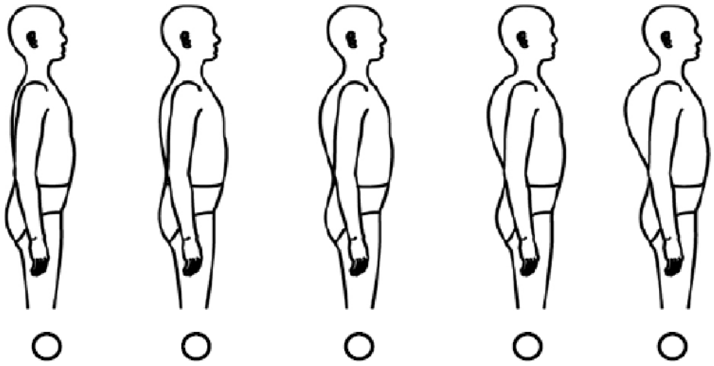

SAQ Expectativas

Quiero estar más equilibrado

- Cierto
- Bastante cierto
- Algo cierto
- Un poco cierto
- Falso

Quiero tener los hombros más equilibrados

- Cierto
- Bastante cierto
- Algo cierto
- Un poco cierto
- Falso

Quiero tener las caderas más equilibradas

- Cierto
- Bastante cierto
- Algo cierto
- Un poco cierto
- Falso

Quiero tener una cintura más equilibrada

- Cierto
- Bastante cierto
- Algo cierto
- Un poco cierto
- Falso
