# Supplementary material for: Body image in idiopathic scoliosis: a comparison study of psychometric properties between four patient-reported outcome instruments
Source: Health Qual Life Outcomes. 2014 Jun 3;12:81. doi: 10.1186/1477-7525-12-81 (PMC4049402; doi:10.1186/1477-7525-12-81)
Supplement: Additional file 2 — Pearson’s correlations among all the scales assessed for the two groups. [file 1477-7525-12-81-S2.pdf]

Pearson's correlations among all the scales assessed for the group < 18 years old

	SAQApp	QLPSDbi	Self-Image SRS-22	SAQ expect	Function SRS22	Pain SRS22	Mental Health SRS22
**TAPS**	-.80**	-.30*	.25	-.44**	.08	.60	.01
**SAQ.App**		.55**	-.51**	.48**	-.40**	-.21	-.15
**QLPSD-bi**			-.81**	.61**	-.50**	-.10	-.46**
**Self-Image SRS-22**				-.60**	.52**	.22	.43**
**SAQ.exp**					-.25	-.16	-.14
**Function srs-22**						.70**	.56**
**Pain SRS-22**							.29

* $\alpha < 0.05$

**$\alpha < 0.01$

Pearson's correlations among all the scales assessed for the group ≥ 18 years old

	SAQApp	QLPSDbi	Self-Image SRS-22	SAQ expect	Function SRS22	Pain SRS22	Mental Health SRS22
**TAPS**	-.71**	-.35*	.53**	-.33	.65**	.44**	.23
**SAQ.App**		.65**	-.76**	.43*	-.64**	-.49**	-.48**
**QLPSD-bi**			-.70**	.63**	-.50**	-.16	-.41*
**Self-Image SRS-22**				-.76**	.72**	.55**	.62**
**SAQ.exp**					-.38*	-.36*	-.30
**Function srs-22**						.76**	.53**
**Pain SRS-22**							.56**

* α< 0.05

**α<0.01
